# Vitamin D Supplementation for the Outcomes of Patients with Gestational Diabetes Mellitus and Neonates: A Meta-Analysis and Systematic Review

**DOI:** 10.1155/2023/1907222

**Published:** 2023-01-14

**Authors:** Chunfeng Wu, Yang Song, Xueying Wang

**Affiliations:** Department of Obstetrics, Shenzhen Longhua Maternity and Child Healthcare Hospital, Shenzhen 51800, China

## Abstract

**Background:**

Prevention and timely treatment of gestational diabetes mellitus (GDM) are important to the prognosis of pregnant women and neonates. We aimed to conduct a meta-analysis to evaluate the effects and safety of vitamin D supplementation on GDM patients and neonates, to provide insights into clinical GDM treatment.

**Methods:**

Two authors searched the Medline, PubMed, Cochrane Library, Web of Science, Embase, CNKI, and Wanfang databases for randomized controlled trials (RCTs) on the effects and safety of vitamin D supplementation in GDM patients. The quality of the included RCTs was evaluated according to Cochrane handbook. RevMan 5.3 software was used for statistical analysis.

**Results:**

A total of 20 RCTs involving 1682 GDM patients were finally included, of whom 837 received vitamin D supplementation. Vitamin D supplementation in GDM patients increased the serum 25(OH)D level (SMD = 4.07, 95% CI: (2.73, 5.41)) and HDL level (SMD = 0.41, 95% CI: (0.23, 0.58)) and reduced serum LDL (SMD = −0.49, 95% CI: (−0.68, −0.29)), TG (SMD = −0.59, 95% CI: (−1.01, −0.17)), and TC (SMD = −0.67, 95% CI: (−1.19, −0.14)) levels in GDM patients (all *P* < 0.05). Besides, vitamin D supplementation reduced the risk of premature birth (OR = 0.37, 95% CI: (0.22, 0.62)), hyperbilirubinemia (OR = 0.38, 95% CI: (0.25, 0.58)), and neonatal hospitalization (OR = 0.38, 95% CI: (0.25, 0.58)) of neonates (all *P* < 0.05). No significant publication bias in synthesized results was found (all *P* > 0.05).

**Conclusions:**

Vitamin D supplementation improves the blood lipid level in GDM patients and reduces adverse neonatal outcomes. The dose and duration of vitamin D supplementation for safety need to be further investigated in future high-quality studies.

## 1. Background

Gestational diabetes mellitus (GDM) is a metabolic disorder, in which glucose tolerance is normal before pregnancy and abnormality occurs for the first time during pregnancy [1]. Previous studies [2–4] have shown that GDM can increase the risk of various perinatal complications, such as hypertensive disorders of pregnancy, polyhydramnios, fetal distress, and preterm birth. Besides, GDM is closely associated with the long-term health impairment of patients and offspring [5]. For example, the incidence of postpartum type 2 diabetes in GDM women is significantly higher than that of normal pregnant women, and the risk of metabolic syndrome in their offspring increases [6]. Previous studies [7, 8] have pointed out that the incidence of GDM has increased significantly in recent years with changes in lifestyle and increasing maternal age. Some studies [9, 10] have pointed out that the incidence of GDM in China is at the middle level, whereas it is at the upper level in the world, and the incidence of GDM is as high as 15.08%. Therefore, the prevention and treatment of GDM are important to the prognosis of pregnant women and newborns.

Studies [11, 12] have shown that vitamin D, as a micronutrient, has a certain correlation with GDM and various adverse maternal and infant outcomes. Studies [13, 14] have shown that in order to maintain the growth of fetal bones during pregnancy, the consumption of vitamin D in pregnant women increases significantly, which can lead to general insufficiency or even deficiency of vitamin D in women after pregnancy. Among them, pregnant women with GDM are a high-risk group of vitamin D deficiency. Supplementation with vitamin D preparations is an important way to prevent vitamin D deficiency during pregnancy. At present, the effect of vitamin D supplementation on GDM is the focus of research by many scholars. There are many domestic and foreign studies on the effects and safety of vitamin D supplementation, but sample sizes are small, and results are not inconsistent. Therefore, this study assessed the effect of vitamin D supplementation on blood lipid levels in GDM patients by conducting a meta-analysis of published randomized controlled trials (RCTs) on the efficacy and safety of vitamin D supplementation in pregnant women with GDM, to evaluate the effects and safety of vitamin D supplementation in GDM women, thereby providing reliable evidence for the treatment of GDM.

## 2. Methods

This meta-analysis and systematic review was conducted and performed according to the preferred reporting items for systematic reviews and meta-analyses (PRISMA) statement [15].

### 2.1. Retrieval Strategy

We searched the Medline, PubMed, Cochrane Library, Web of Science, Embase, CNKI, and Wanfang databases for RCTs on the effects and safety of vitamin D supplementation in pregnant women with GDM. The search date limit of databases was from the establishment of the database to May 15, 2022. The search formula used in this meta-analysis was (vitamin D OR 25-(OH)D OR 1.25(OH)2D) AND (gestational diabetes OR GDM OR diabetes and pregnancy). In addition, we performed additional searches for the references of the included RCTs and relevant reviews to make literature research more comprehensive.

### 2.2. Inclusion and Exclusion Criteria for RCTs

The inclusion criteria for this meta-analysis were as follows: (1) RCT study design, the published language was Chinese or English; (2) patients diagnosed with GDM according to clear diagnostic criteria; (3) the intervention group was supplemented with vitamin D, and the control group was supplemented with placebos or without vitamin D supplementation; (4) relevant data could be extracted. The exclusion criteria for this meta-analysis were as follows: (1) the types of literature studies were case reports and reviews; (2) articles with repeated reports; and (3) the data on outcomes could not be extracted.

### 2.3. Literature Quality Evaluation

Two researchers independently completed quality evaluation and data extraction, then cross-checked the work, and discussed and resolved any disagreements. The quality of the included studies was evaluated in accordance with the evaluation criteria recommended by the Cochrane Systematic Review Guidebook [16]. Evaluation contents mainly included the following seven aspects: generation of random sequences, concealment of assignment sequence, blinding of all study participants and personnel, blinding of outcome assessments, completeness of outcome data, selective outcome reporting, and other sources of bias. Every item could be rated “yes,” “no,” or “unclear” accordingly.

### 2.4. Data Extraction

Two authors screened the identified articles and extracted data accordingly. The data extraction content of this meta-analysis included first author, publication time, region, age, GDM diagnostic criteria, vitamin D testing method, sample size, intervention details of intervention and control groups, and study outcome indicators. Outcomes extracted from this meta-analysis were serum 25-hydroxyvitamin D levels, total cholesterol (TC), low-density lipoprotein cholesterol (LDL), high-density lipoprotein cholesterol (HDL), triglycerides (TG), incidence of hyperbilirubinemia, premature birth, and neonatal hospitalization.

### 2.5. Statistical Analysis

This meta-analysis used RevMan 5.3 software for statistical analysis. We tried to transform and uniform the units of vitamin D measurement to make results consistent. The standardized mean difference (SMD) and the odds ratio (OR) were used to calculate effect statistics and the 95% confidence interval (CI), and the chi-square test (test level was 0.1) was used to evaluate heterogeneity. When the homogeneity of the research results was good (*P* > 0.1, when *I*^2^ < 50%), a fixed-effect model was used; otherwise (*P* ≤ 0.1, when *I*^2^ ≥ 50%), a random-effect model was used. In addition, pooled effect sizes were re-estimated after excluding individual studies in turn, and data were reanalyzed using different statistical methods to test the sensitivity of the results. We used funnel plot symmetry to judge whether there was publication bias, and we performed Egger regression analysis to evaluate the publication bias of the literature. In this meta-analysis, *P* < 0.05 was considered to be statistically significant between groups.

## 3. Results

### 3.1. Study Selection and Characteristics

**Figure 1:**
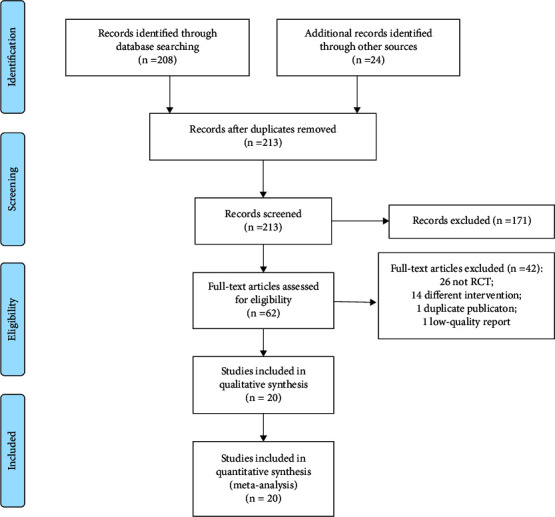
PRISMA flow diagram of study selection.

As indicated in Figure 1, 232 studies were initially identified, and after filtering layer by layer, a total of 20 RCTs [17–36] were included. Of the 20 included RCTs, a total of 1682 patients were involved, of whom 837 received vitamin D supplementation. The characteristics of the included RCTs are presented in Table 1.

### 3.2. Quality of Included RCTs

The quality assessment of the literature included in this meta-analysis is shown in Supplementary Figures 1 and 2. All the included RCTs adopted the principle of randomized control, data integrity was good, and there was no other bias. However, some RCTs [27, 28, 30, 34, 35] did not explain the concealment of the allocation sequence and the use of the blinding method. All RCTs used internationally certified standard methods to measure outcome indicators, and some studies were lost to follow-up.

### 3.3. Meta-Analysis

#### 3.3.1. Serum 25(OH)D Level

**Figure 2:**
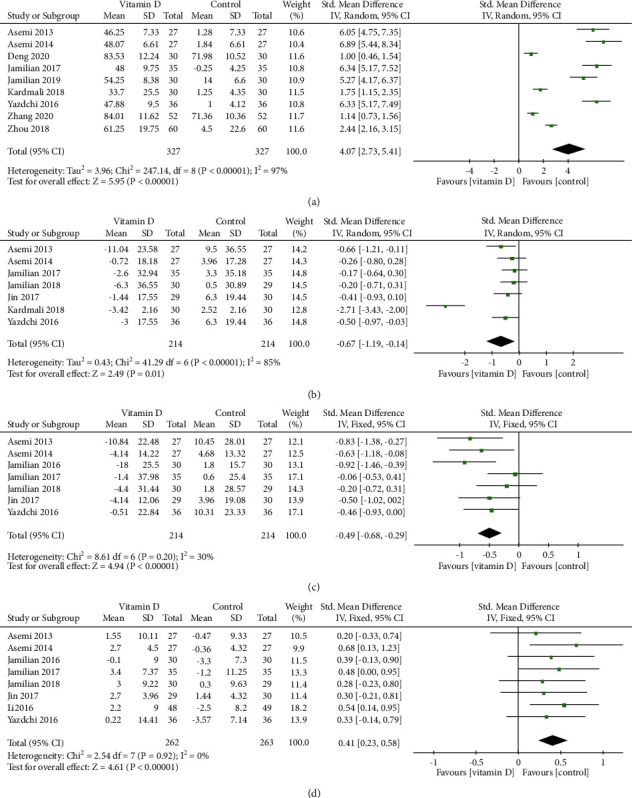
Forest plots for (a) the serum 25(OH)D level, (b) the serum TC level, (c) the serum LDL level, and (d) the serum HDL level.

Nine RCTs [17, 18, 20, 23, 24, 26, 27, 35, 36] reported the serum 25(OH)D level. There was statistical heterogeneity among the analyzed data (*I*^2^ = 97%, *P* < 0.001), so a random-effect model was used for the meta-analysis, and the results showed that vitamin D supplementation intervention could significantly increase serum 25(OH)D levels in GDM patients (SMD = 4.07, 95% CI: (2.73, 5.41), *P* < 0.001, Figure 2(a)).

#### 3.3.2. Serum TC Level

Seven RCTs [17, 18, 21, 24, 26, 29, 35] reported the serum TC level. There was statistical heterogeneity among the analyzed data (*I*^2^ = 85%, *P* < 0.001), so a random-effect model was used for the meta-analysis, and the results showed that vitamin D supplementation intervention could significantly reduce the TC levels in GDM patients (SMD = −0.67, 95% CI: (−1.19, −0.14), *P*=0.01, Figure 2(b)).

#### 3.3.3. Serum LDL Level

Seven RCTs [17, 18, 21, 22, 24, 29, 35] reported the serum LDL level. There was no statistical heterogeneity among the analyzed data (*I*^2^ = 30%, *P*=0.20), so a fixed-effect model was used for the meta-analysis, and the results showed that vitamin D supplementation intervention could significantly reduce the LDL levels in GDM patients (SMD = −0.49, 95% CI: (−0.68, −0.29), *P* < 0.001, Figure 2(c)).

#### 3.3.4. Serum HDL Level

Eight RCTs [17, 18, 22, 24, 26, 29, 30, 35] reported the serum HDL level. There was no statistical heterogeneity among the analyzed data (*I*^2^ = 0%, *P*=0.92), so a fixed-effect model was used for the meta-analysis, and the results showed that vitamin D supplementation intervention could significantly increase the HDL levels in GDM patients (SMD = 0.41, 95% CI: (0.23, 0.58), *P* < 0.001, Figure 2(d)).

#### 3.3.5. Serum TG Level

**Figure 3:**
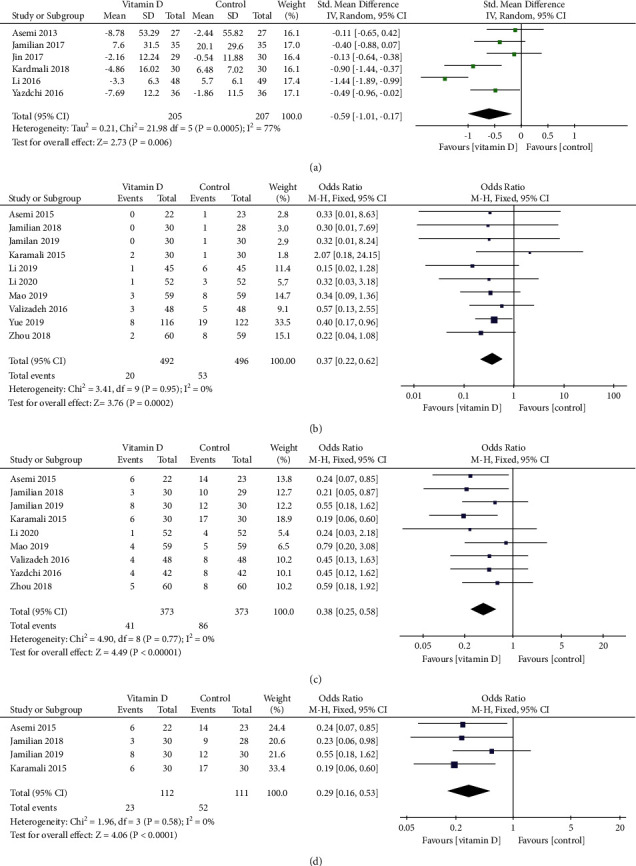
Forest plots for (a) the serum TG level and the incidence of (b) premature birth, (c) hyperbilirubinemia, and (d) neonatal hospitalization.

Six RCTs [17, 24, 26, 29, 30, 35] reported the serum TG level. There was no statistical heterogeneity among the analyzed data (*I*^2^ = 77%, *P* < 0.01), so a random-effect model was used for the meta-analysis, and the results showed that vitamin D supplementation intervention could significantly reduce the TG levels in GDM patients (SMD = −0.59, 95% CI: (−1.01, −0.17), *P*=0.006, Figure 3(a)).

#### 3.3.6. Incidence of Premature Birth

Nine RCTs [19–21, 23, 31–33, 35] reported the incidence of premature birth. There was no statistical heterogeneity among the analyzed data (*I*^2^ = 0%, *P*=0.77), so a fixed-effect model was used for the meta-analysis, and the results showed that vitamin D supplementation intervention could significantly reduce the incidence of premature birth in neonates (OR = 0.38, 95% CI: (0.25, 0.58), *P* < 0.001, Figure 3(b)).

#### 3.3.7. Incidence of Hyperbilirubinemia

Ten RCTs [19–21, 23, 28, 31–34] reported the incidence of hyperbilirubinemia. There was no statistical heterogeneity among the analyzed data (*I*^2^ = 0%, *P*=0.95), so a fixed-effect model was used for the meta-analysis, and the results showed that vitamin D supplementation intervention could significantly reduce the incidence of hyperbilirubinemia in neonates (OR = 0.37, 95% CI: (0.22, 0.62), *P* < 0.001, Figure 3(c)).

#### 3.3.8. Incidence of Neonatal Hospitalization

Four RCTs [19, 21, 23, 25] reported the incidence of neonatal hospitalization. There was no statistical heterogeneity among the analyzed data (*I*^2^ = 0%, *P*=0.58), so a fixed-effect model was used for the meta-analysis, and the results showed that vitamin D supplementation intervention could significantly reduce the incidence of neonatal hospitalization (OR = 0.29, 95% CI: (0.16, 0.53), *P* < 0.001, Figure 3(d)).

### 3.4. Sensitivity Analysis and Publication Bias

We sequentially excluded individual studies for sensitive analysis to evaluate the stability of the results. The results showed that combined effect values before and after the exclusion of any study were relatively close, and the study results did not change significantly, suggesting that the results of each meta-analysis were stable.

**Figure 4:**
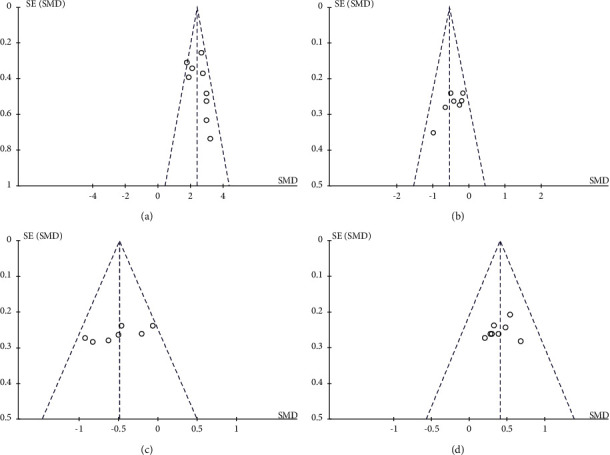
Funnel plots for (a) the serum 25(OH)D level, (b) the serum TG level, (c) the serum LDL level, and (d) the serum HDL level.

**Figure 5:**
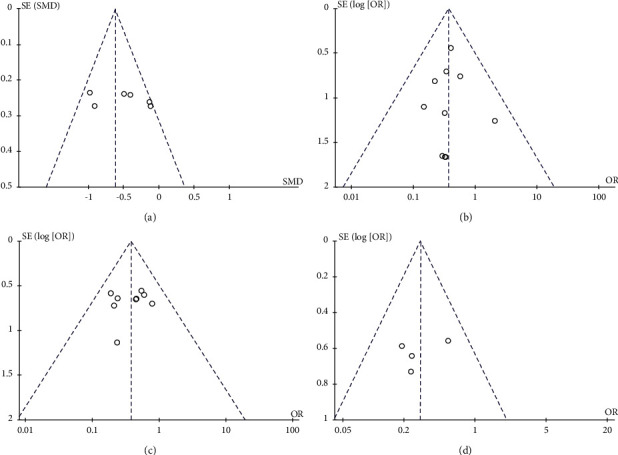
Funnel plots for (a) the serum TG level and the incidence of (b) premature birth, (c) hyperbilirubinemia, and (d) neonatal hospitalization.

The distribution of points on the funnel plot of each variable was symmetrical (Figures 4 and 5). The results of Egger regression analysis indicated that there was no significant publication bias in the results of each meta-analysis (all *P* > 0.05).

## 4. Discussion

GDM is the most common complication of pregnant women during pregnancy, and prevalence has gradually increased in recent years. The probability of type 2 diabetes, metabolic syndrome, and obesity in GDM patients and their offspring can be as high as 60.16% [37, 38]. The pathogenesis of GDM has not yet been elucidated. Some studies [39, 40] suggest that the occurrence and development of GDM are closely related to dietary structure, family history of diabetes, obesity, chronic inflammatory response, genetic differences, insulin resistance, pancreatic *β*-cell damage, and immune dysfunction. In recent years, in order to prevent the occurrence of gestational diabetes mellitus, clinical blood glucose monitoring is usually carried out according to the pregnancy cycle of pregnant women. However, it is mostly detected at 24 to 28 weeks of pregnancy. The treatment of GDM at this stage is more difficult and may have caused harm to health of mothers and babies [41]. Therefore, clinical diagnosis of gestational diabetes mellitus should be performed as soon as possible, and targeted treatment should be given to avoid adverse pregnancy outcomes. The results of this present meta-analysis have shown that vitamin D supplementation is beneficial to increasing the serum 25(OH)D and HDL levels and is helpful for reducing the serum TC and LDL levels of GDM patients and maternal hyperbilirubinemia as well as neonatal hyperbilirubinemia and hospitalization risk. There are some discrepancies between the results of this meta-analysis and other previous meta-analyses [42, 43]. Previous meta-analyses [42] have found that vitamin D can improve LDL levels, but they did not find its effects on TG, TC, and HDL. The possible reason for this is that most of the included RCTs have an intervention time of less than 6 weeks, and there is a lack of long-term follow-up studies. Multiple studies [44–46] have shown that when GDM patients have abnormal lipid metabolism, their risk of pregnancy complications increases. Therefore, vitamin D supplementation is of great significance for improving the prognosis of GDM patients and neonates, and it is worthy of clinical promotion and use for GDM treatment.

Vitamin D is a hormone-like substance, which can promote the secretion of insulin in the human body under normal physiological conditions and promote normal glucose tolerance in the body, and it can effectively regulate the content of calcium ions in the body [47]. The deficiency of vitamin D is closely related to the occurrence of gestational diabetes mellitus. By detecting the content of vitamin D in pregnant women with gestational diabetes mellitus, the degree of deficiency can be clarified and a reasonable supplementation plan can be formulated as soon as possible. For pregnant women with GDM who are overweight or obese before pregnancy, diet and weight should be strictly controlled and blood sugar management should be strengthened. Previous studies [48–50] have pointed out that vitamin D can regulate insulin secretion through the following pathways: First, vitamin D affects the function of pancreatic islet B cells by directly activating VD receptors or by interfering with VD response elements in the insulin receptor-initiating gene region; second, vitamin D improves insulin sensitivity and glucose transport by enhancing the response of insulin receptors to insulin; third, vitamin D increases the conversion of proinsulin to insulin. In addition, it has been reported that active vitamin D can reduce food intake, reduce body weight, and improve glucose tolerance and insulin sensitivity through vitamin D receptors in the paraventricular nucleus of the hypothalamus.

Vitamin D is a fat-soluble vitamin that plays an important role during pregnancy. In recent years, many studies [51, 52] have suggested that vitamin D is closely related to GDM. Animals with vitamin D deficiency (especially in early life) have an increased incidence of diabetes, and supplementation of vitamin D and its analogs can reduce or delay the occurrence of diabetes [53]. Studies [54, 55] have shown that vitamin D deficiency is associated with an increased incidence of type 2 diabetes, and vitamin D supplementation can significantly increase insulin sensitivity in people with insulin resistance and vitamin D deficiency. Insulin resistance and insufficient secretion are one of the pathogeneses of GDM [56]. Vitamin D levels are negatively correlated with blood sugar, and they are positively associated with insulin resistance. Vitamin deficiency in pregnant women with GDM increases the risk of insulin resistance and metabolic syndrome [57, 58]. At present, an international consensus has not been reached on the dosage of VD supplementation during pregnancy. The dietary nutrient reference amount for Chinese residents recommends a routine vitamin D supplementation of 400 U/d during pregnancy, and the maximum tolerated dose is 2000 U/d. At present, most experts believe that 1000–2000 U/d can be supplemented for pregnant women with vitamin D deficiency during pregnancy, and the maximum safe dose is 4000 U/d. However, the dose and safety of vitamin D supplementation during pregnancy remain to be further studied in the future.

Neonatal hyperbilirubinemia is a common yet serious clinical disease, which damages the nervous system of infants and young children, resulting in sequelae such as involuntary movements of hands and feet, deafness, and even cerebral palsy with serious long-term damage [59–61].

This meta-analysis has found that vitamin D supplementation during pregnancy in mothers with GDM reduces the incidence of hyperbilirubinemia, preterm birth, and neonatal hospitalization. The possible reason is that vitamin D deficiency is prevalent in pregnant women, and vitamin D supplementation can increase the formation of antimicrobial peptides in the body, inhibit the production of inflammatory cytokines, and play an important role in immune regulation [62]. In addition, studies [63, 64] have shown that vitamin D deficiency during pregnancy is associated with preterm birth and hospitalization rates of neonates. Some studies [65–67] have pointed out that vitamin D supplementation can improve the maternal vitamin D status during pregnancy. The improvement of maternal vitamin D status may directly affect the fetal vitamin D supply and neonatal vitamin D level, thereby reducing the risk of preeclampsia and premature birth [68]. Therefore, vitamin D supplementation during pregnancy is very important and necessary for the prognosis of pregnant women and neonates.

There are some limitations in this meta-analysis worth considering. First, most of the included RCTs are from China and Iran, which may have certain geographical or population bias. Second, the study design of group concealment and outcome blinding in some included RCTs is not rigorous, and there can be certain biases in outcomes. Third, the weight gain during pregnancy may be a confounding factor for our results, yet we failed to conduct subgroup analysis based on the weight gain during pregnancy as limited by the collected data. Finally, the heterogeneity of the synthesized results of some outcome indicators is high, which may be related to the differences in the dose and treatment duration of vitamin D included in RCTs. Therefore, the therapeutic effect and safety of vitamin D in GDM patients still need to be further explored in future large-sample, strictly designed, high-quality studies.

## 5. Conclusions

In conclusion, the results of this meta-analysis have found that vitamin D supplementation during pregnancy in GDM patients can reduce serum LDL, TG, and TC levels and increase the serum 25(OH)D level and HDL level in GDM patients. Besides, vitamin D supplementation is beneficial to reducing maternal hyperbilirubinemia, as well as neonatal hyperbilirubinemia and hospitalization risk. Vitamin D supplementation can effectively improve the prognosis of pregnant women with GDM and reduce the incidence of adverse pregnancy outcomes. It is worth noting that the dose and duration of vitamin D supplementation still need to be further analyzed and investigated in future high-quality studies to provide evidence for the prevention and quality of GDM.

## Figures and Tables

**Table 1:** Characteristics of the included RCTs.

RCT	Country	Age (y)	Sample size		Detection method	Intervention		Duration of follow-up (weeks)
			Vitamin D group	Control group		Vitamin D group	Control group	
Asemi 2013	Iran	31.5 ± 6.1	27	27	ELISA	50,000 U VD_3_/21 d, 2 times/day	Placebo	6
Asemi 2014	Iran	18∼40	28	28	ELISA	VD_3_ 50,000 U/2 week	Placebo	6
Asemi 2015	Iran	30.9 ± 5.8	22	23	ELISA	50,000 U VD_3_/21 d, 2 times/day	Placebo	6
Deng 2020	China	18∼35	30	30	ELISA	400 U VD_3_/d	Placebo	8
Jamilian 2016	Iran	28.4 ± 6.2	30	30	ELISA	1000 U VD_3_/d	Placebo	6
Jamilian 2017	Iran	18∼40	35	35	ELISA	VD_3_ 50,000 U/2 week	Placebo	6
Jamilian 2018	Iran	18∼35	30	28	ELISA	VD_3_ 50,000 U/2 week	Placebo	6
Jamilian 2019	Iran	18∼35	30	30	ELISA	200 U VD_3_/d	Placebo	6
Jin 2017	China	18∼35	29	30	ELISA	2000 U VD_3_/d	Black control	6
Karamali 2015	Iran	18∼40	30	30	ELISA	50,000 U VD_3_/21 d, 2 times/day	Placebo	6
Karamali 2018	Iran	18∼40	30	31	ELISA	200 U VD_3_/d, 2 times/day	Placebo	6
Li 2016	China	28.0 ± 4.0	48	49	ELISA	500 U VD_3_/d	Placebo	16
Li 2019	China	35.2 ± 5.2	45	45	ECL	400 U VD_3_/d	Black control	6
Li 2020	China	20∼40	52	52	ELISA	400 U VD_3_/d	Placebo	6
Mao 2019	Iran	18∼35	59	59	ELISA	400 U VD_3_/d	Placebo	8
Valizadeh 2016	Iran	32.0 ± 5.0	48	48	ELISA	700,000 U VD_3_ in total	Placebo	5
Yazdchi 2016	Iran	31.9 ± 4.0	36	36	ECL	VD_3_ 50,000 U/2 week	Placebo	8
Yue 2019	China	18∼35	116	122	ECL	1200 U VD_3_/d	Placebo	16
Zhang 2020	China	25∼30	52	52	ELISA	400 U VD_3_/d	Placebo	2
Zhou 2018	China	18∼35	60	60	ECL	400 U VD_3_/d	Black control	16

## Data Availability

All data generated or analyzed during this study are included in this published article.

## Supplementary Materials

Supplementary Materials Supplementary Figure 1: risk of the bias graph. Supplementary Figure 2: risk of bias summary.
