# Supplementary figures and images for: Vitamin D Supplementation for the Outcomes of Patients with Gestational Diabetes Mellitus and Neonates: A Meta-Analysis and Systematic Review
Source: Int J Clin Pract. 2023 Jan 14;2023:1907222. doi: 10.1155/2023/1907222 (PMC9867594; doi:10.1155/2023/1907222)

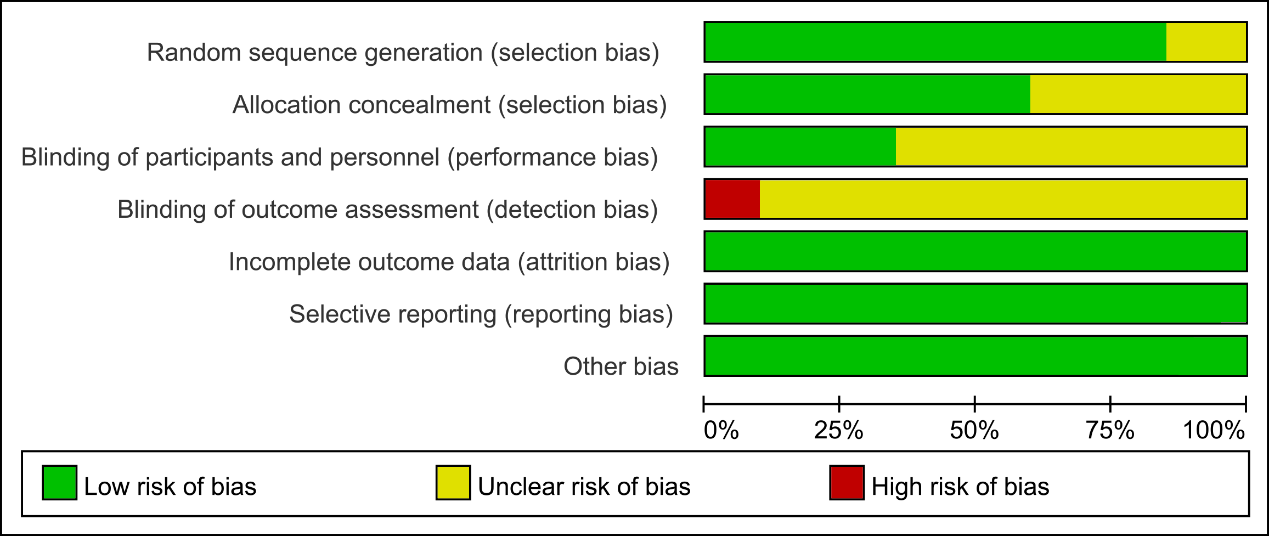
Supplementary figure 1 Risk of bias graph


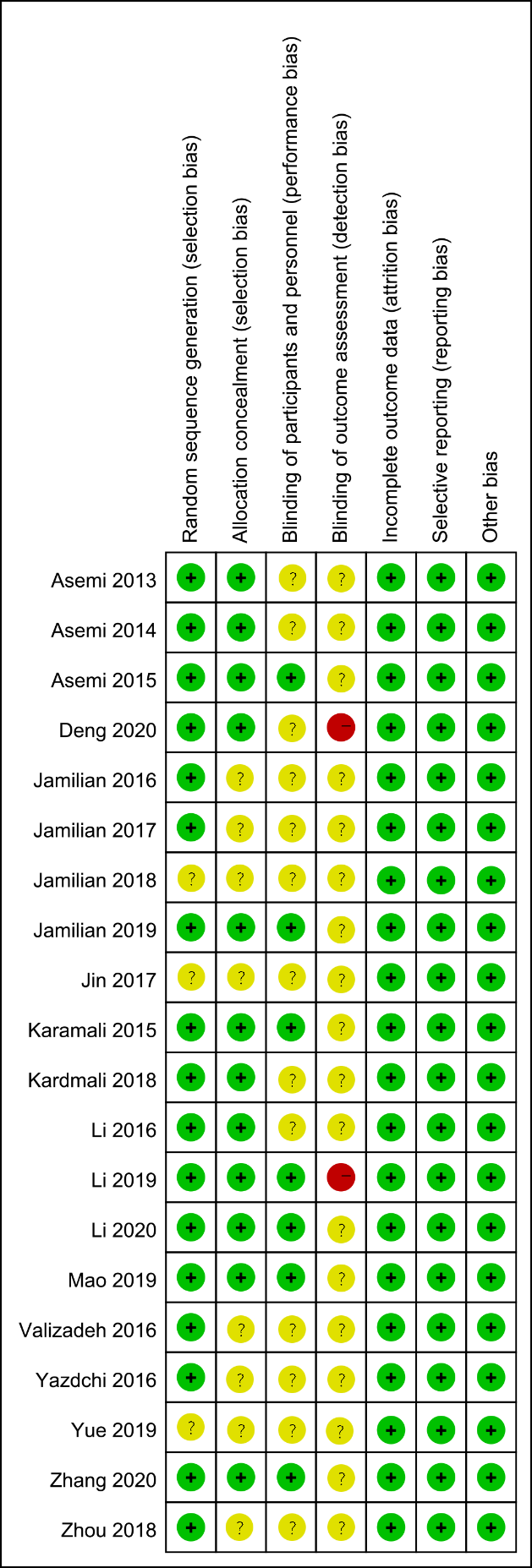


Supplementary figure 2 Risk of bias summary

Supplement: Supplementary Materials — Supplementary Figure 1: risk of the bias graph. Supplementary Figure 2: risk of bias summary. [file 1907222.f1.docx]
